# Machine learning in oncology: a review

**DOI:** 10.3332/ecancer.2020.1065

**Published:** 2020-06-30

**Authors:** Cecilia Nardini

**Affiliations:** European School of Molecular Medicine (SEMM), 20139 Milan, Italy

**Keywords:** machine learning, big data, methodology, ethics, image recognition, deep learning, neural network

## Abstract

Machine learning is a set of techniques that promise to greatly enhance our data-processing capability. In the field of oncology, ML presents itself with a wealth of possible applications to the research and the clinical context, such as automated diagnosis and precise treatment modulation. In this paper, we will review the principal applications of ML techniques in oncology and explore in detail how they work. This will allow us to discuss the issues and challenges that ML faces in this field, and ultimately gain a greater understanding of ML techniques and how they can improve oncological research and practice.

## Introduction

Over the course of the last two decades, we have witnessed a progressive spread of machine learning (ML) techniques and applications. From shopping recommendation software to sophisticated image and speech recognition systems, ML is present in our lives in a quietly intrusive manner. For the scientist and medical practitioner, the presence of ML techniques is felt also in the workplace: in the wealth of algorithms and ML-based tools that aid, and in some cases have come to supersede, the human practice in the biomedical sciences.

‘ML’ is an umbrella term for a set of computational techniques that permit computer systems to ‘learn’, i.e., to become progressively better over time at performing a given task. Its approach to learning is ‘brute-force’ in the sense that it is based upon a huge amount of data that the computer system runs through in a trial-and-error process in order to minimise the deviation of its prediction from an expected result.

The general developments in computing and digital technology that enabled the progress in ML techniques, at the same time also greatly enhanced the data-acquisition and data-storage capabilities in several fields of scientific research, bringing about the so-called ‘Big Data’ age. The unprecedented availability of data, in turn, allowed ML techniques to be successfully employed in these domains. From techniques that facilitate diagnosis by revealing complex patterns in screening data, to expert systems that make treatment decisions, ML is becoming increasingly pervasive in the medical practice and oncology is no exception to the trend.

In this paper, we will review the principal applications of ML techniques in oncology, both in the clinical practice and in the drug research area. A detailed explanation of these applications, and of how they work, will allow us to discuss the issues and challenges that ML faces in this field with a heightened level of awareness, and ultimately gain a greater understanding of ML techniques and how they can improve oncological research and practice.

## Different kinds of learning

As outlined in the introduction, ML is a set of techniques for training computational systems to perform certain knowledge-related tasks. The actual techniques may vary depending on the specific task to be learned, but a look at a specific case is useful in order to flesh out the general principle. To this aim, we will consider one of the best-known ML techniques, image recognition.

Suppose we want to train a computer system to recognise cars. In order to do this, the computer system is presented with some images and has to output a yes/no answer (contains a car or not). Clearly, at the beginning of this training the system has no way of distinguishing cars, so the first answers will be at random. However, at this point, it will be possible to feed the right answers back onto the computer system, which will use this knowledge to re-adjust its internal parameters in order to commit fewer mistakes the next time around. The ML algorithm is precisely the rule that the algorithm follows to update its internal parameters in the face of a discrepancy between its prediction and the actual result.

When this process is repeated a large enough number of times, eventually the computer system will ‘converge’, i.e., it will recognise correctly all the cars within the images that have been used to train it. However, what about cars in new images, ones that the system has never ‘seen’? If the training set is sufficiently large (and ‘large’ generally means tens or hundreds of thousands of annotated images), the system will have captured the defining features of a car and thus it will be able to recognise cars in images that it has never seen. The accuracy of the most sophisticated ML systems in this kind of image recognition tasks is very high, rivalling and possibly even surpassing human capacities.

Image recognition is an instance of a *supervised learning* task. This means that the system is trained using a set of labelled data, i.e., data where the feature to be learnt is known and the knowledge is passed on to the system via feedback during training—in the previous example, the images have an embedded reference to the information about whether they represent a car or not. Supervised learning is contrasted with *unsupervised learning* techniques, where the data used in the training are unlabelled. The system has to learn how to classify or structure the data without any prior knowledge of what the groups or structures may be, but simply by trying in an iterative manner different ways to organise the data and selecting the one that is most stable or that minimises discrepancy according to a chosen criterion. Unsupervised learning is generally not employed to do visual recognition but, in keeping with our example of a car recognition task, the analogy is as follows. In this case, we would submit to the computer system a vast set of unlabelled images and have the system classify them. If some images contain cars while others do not, over many repetitions the system will learn that classifying images with ‘car features’ together leads to a more successful classification (i.e., one that is more likely to be repeated next time around) and, in this way, it will learn to identify ‘car features’ also in novel images.

ML algorithms used in oncology belong to both families, as we will shortly see. There is a further kind of ML task, so-called *reinforcement learning* tasks, where the system has to learn how to achieve a certain goal in a dynamic environment. In this case, there is no correct or best answer and the system learns from the feedback it receives from the environment. This latter family of techniques is for instance used to train systems to play games or drive cars, and at present it does not have an application in the biomedical context.

ML techniques can be further characterised in terms of the underlying model: every ML application is based on a model, which defines the mathematical structure of the intelligent system and the way it learns. Some models can be used only for a certain kind of task. For instance, the various clustering models (*k*-means clustering or Gaussian mixture) are used for unsupervised learning; some classification models (like Support Vector Machines) or regression models (e.g., decision trees) are used for supervised learning. Other kinds of models are more versatile and can be adapted to tackle a wide array of problems both in the supervised and unsupervised learning field. An example is Artificial Neural Networks, a class of models inspired by the structure and functioning of biological brains of which deep learning network models provide a special case.

In the following section, we will review, by means of some representative examples, the application of these techniques to oncological practice both in the lab and in the clinics. This will allow us to enter the discussion of the merits and issues of these techniques with a clear picture in mind.

## Applications to oncology

Supervised learning techniques, such as image recognition, are often employed in tumour diagnosis and grading or staging, since this kind of problem is equivalent to assigning imaging samples to known categories. For example, a ML system may learn how to recognise particular structures in mammograms that are associated with higher breast cancer risk [19] or perform Gleason scoring of prostate cancers [12] or classify skin cancers based on the visual appearance of lesions [7].

Another area of great interest is the prediction of disease progression or treatment effectiveness. In this case, the kind of structure or feature that the system is trained to recognise is more often an abstract quantity rather than a visual image: the result of genomic or molecular analyses, such as gene expression profiles, microarray analysis or PCR (see Kourou *et al* [16] for a critical review). An example is prognostic classification of B-cell lymphoma [17]: the algorithm learns to recognise features in the patient’s gene expression profile that allows classification into one of two groups with very different prognosis (survival rates).

This is indeed an interesting application of ML. Molecular and genomic techniques have become increasingly crucial to the oncological practice, in that they can provide unique information on disease diagnosis, classification, progression and response to treatment; however, the results of these analyses are often difficult to interpret, even with the help of various human-friendly visualisation techniques (such as heat maps) that have been developed over the years. Computer systems do not have the same cognitive limitations as humans, and therefore they can be trained to recognise a characteristic profile or ‘fingerprint’ in this kind of data in the same manner as they can be trained to recognise features in visual images. (It is actually the other way around. Computers do not ‘see’ images in the human sense of the term: pictures and other visual records are processed by an image recognition algorithm in the form of digital records. Therefore, visual recognition for a computer is really just a special case of pattern recognition in numeric data.) The advantage of employing ML tools for this kind of task is obvious; however, it should be noted that ML can bear an advantage also in the case of purely visual data, in that it can reduce error, bias and the interpersonal variability that go with this kind of tasks in the clinics [18].

Unsupervised learning algorithms typically find an application where large annotated datasets to be used for supervised learning are not available or when the features of interest are not known. In this case the ML system will be fed a large number of patient data (for instance microarrays, gene expression profiles or histopathological or imaging data) but, unlike with supervised learning techniques, the data will be unlabelled, so the emerging classification will be done on features that are not known in advance. For instance, Lynch *et al* [11] explore different clustering techniques on simple disease variables (such as age of the patient, tumour stage, tumour size etc.) to automatically classify patients into groups with different survival rates. This application is unsupervised because the researchers do not know in advance that a particular combination of, for instance, grade and number of primaries will be associated with a better prognosis: the resulting classification will emerge from the learning system’s analysis of the data.

The stratification of patients according to phenotypical characteristics of their tumour or other biological markers, in order to derive some subtyping or categorisation with prognostic or predictive value, is a crucial problem in so-called *personalised* or *precision* Oncology. The precision approach to Medicine consists in taking into account disease and personal variability to predict more accurately which treatment and prevention strategies will work for a particular disease in a specific group of people. In Oncology, it is now known that cancers that were once treated as a single disease (such as lung or breast cancer) are actually very different diseases when considered in light of their histopathological and molecular detail (e.g., small cell versus squamous cell carcinoma, or HER2-Positive versus triple negative breast cancer), and this difference reflects in prognosis and in the appropriate treatment choice. In this context unsupervised ML techniques, particularly clustering algorithms, can be used for in-depth and novel classification of disease and patients groups that may be difference-makers in drug response or predicted survival [1, 3]. Another area of application is the discovery of new drugs or biomarkers [8]: here unsupervised techniques can be useful for a first coarse-grained exploration of the space of possible candidates to help identify avenues for further investigation.

A more extreme example of the power of these techniques is the use of ML methods to extract knowledge and understanding from the impressive deluge of -omic data that are available today, such as stem from projects like the The Cancer Genome Atlas (e.g., Ciriello *et al* [4]). It is in these kinds of challenges that unsupervised learning appears most attractive as a technology in that it appears to generate new knowledge from otherwise hard to interpret data; this capacity, however, comes with specific caveats and pitfalls that will be explored in the next section.

## Machines that learn: some issues

The use of ML methods represents a novel approach in the clinical and research setting and as such it brings about some specific issues and problems. However, it is also true that many of the methodological caveats and concerns in the use of such techniques are by no means unique to them, but are common to all complex technologies used in biomedical practice. In this section, we will examine both families of issues. The ideas discussed in this section are not specific to the oncological context, unless otherwise specified, but are common to all applications of ML to biomedical disciplines.

A worry that often comes to the forefront when discussing ML is related to the fact that the algorithms performing ML recognition and decision tasks are often referred to as ‘expert systems’. This definition evokes a picture of the ML algorithm as an artificial agent with decision power over human lives and it fosters the idea that such systems have come to replace human judgement. However, even when employed in a diagnostic or treatment adjustment capacity, these algorithms always act only as a support tool for a decision taken by a human doctor and for which a human doctor will bear the ultimate responsibility. While it is possible to envisage that in the near future the human role in some of these tasks will be entirely superseded, this will most likely never happen pending a clarification of who will be held accountable for the expert system’s decisions and possible errors. The case of self-driving cars represents a good example: development of this technology has at present mostly stalled because there is no clear idea of how traffic regulation laws could fit self-driving systems.

While it is at present an exaggeration to worry about learning systems becoming a replacement for human specialists, it is, however, true that the mechanism producing the results in a ML algorithm often appears opaque. There is a fear that, since the human doctor will in most cases be unable to reconstruct the reasoning or the evidence supporting the digital system output, this output, upon which a diagnostic or treatment decision will be based, will appear as black-boxed piece of information that the doctor has to take as-is, or discard entirely. It is, however, possible to argue that, to an extent, this is already happening with many technological tools and techniques on which medical professionals have come to rely over the years, and the difference, if any, is one of degree more than of substance. For instance, today’s oncological practice is supported by many technologies, such as biomarker assessment or genomic profiling, for which the connection with the oncological disease is rather obscure to the non-specialist. Confidence in these technologies has been built through results, such as proven successful predictions, and the same can be true of ML algorithms. In other words, the oncologist can rely on a tumour diagnosis provided by an algorithm if the algorithm has been proven to yield correct diagnoses in most cases with consistency, in the same way as they rely on the value of a certain biomarker to be a proxy for tumour remission or on the result of a genomic assay to be an indicator of the most appropriate treatment course.

Considering ML as a tool not dissimilar to the others helping the clinician in the lab or in the operating room is useful also to illuminate a second family of issues, more methodological in nature that arise specifically in connection with ML techniques. To better understand what they are it is useful to consider the two families of ML techniques—supervised and unsupervised—in turn.

In supervised learning, the algorithm is trained through exposure to a set of labelled inputs and it ‘learns’ how to associate some features in the input with a label. A couple of things can go wrong in building this kind of association: some of the training input might be mislabelled, more obviously; or, more subtly, the training set might be biased in some way and such bias may inadvertently be amplified by the trained system. For example, if a feature unrelated to the tumour presence is consistently present in imaging screens of tumours used to train a diagnostic system (for instance a ruler or grid in skin cancer images), the system will learn to consider said feature as a predictor of tumour presence even though the association is false. This worry is of great relevance especially for visual recognition algorithms: due to the huge amount of data needed to train this kind of systems, and due to the fact that even moderately large annotated datasets of tumour imaging are hard to come by, often such systems are trained for the most part using some large general purpose image recognition dataset and then receive a more specialised training on a smaller task-specific labelled training set. It is clear that in this manner the possibility for the system to learn spurious correlations is very high. A related but separate issue is the possibility that if, as it often happens, a certain demographic is disproportionately represented in the training set, the algorithm may perform poorly on other patient groups [14].

In unsupervised learning techniques, on the other hand, the input set on which the training happens is composed of unlabelled, unorganised data. In the unsupervised learning case, it is the algorithm’s task to identify a correlation or a structure in the input, without any previous label assignment by the human. In this case mislabelling or misattribution is not an issue. Instead, unsupervised techniques are subject to the same kind of issues as may be familiar to the user of classical statistical tools. Due to the nature of the technique, the most cogent among these is *overfitting*, or, excessive adherence to the training data. In unsupervised learning, the computer system learns by maximising its performance over some metrics upon the training set. Theoretically, it does so by extracting a meaningful, generalisable pattern that can then be applied to new observations. However, the same result of a good fit with the training set can be obtained simply by ‘memorising’ the training set, similar to how it is possible to cheat at a test if one knows the answers in advance. Clearly, in this case, the actual predictive power of the model on new data will be poor; however, this can be difficult to ascertain for real-world applications.

An important point to clarify, before moving on to the discussion, concerns the difference between ML and techniques of traditional statistical analysis. At first glance, some unsupervised ML techniques bear a strong resemblance with statistical techniques, to the point that they might appear simply as a high-power version of the same thing. For example, some forms of clustering analysis can be considered a more powerful version of principal components analysis. The point of the similitude (and the difference) between ML and statistics has been analysed at depth in a recent Nature Methods article [2] and the main difference is purported to be in the application of the two techniques: classical statistical methods are mainly used for inference, i.e., to detect relations among the data; ML methods are predominantly used for prediction, i.e., to guide decision in specific cases. This conclusion underscores the idea that the knowledge generated by traditional statistical models is more robust and closer to capturing ‘true’ physiopathological effects. This is relevant to the potential use of ML techniques in clinical trials and other regulatory settings, and it will be explored in the next section.

## ML to the trial

As we have seen in the preceding sections, ML algorithms represent a promising tool in the clinics. They can tackle simple clinical questions with an ability similar, or superior, to human pathologists. Trained on thousands of tumour images, they embed years of clinical experience, comparable to the most seasoned expert. They can detect patterns and meaningful classifications in otherwise intractable genomic and molecular data. However, they also have limitations, and their successful application is not so straightforward.

At this point, in the discussion, it is possible to address an important methodological question: are ML techniques at their present state adequate to complement or replace existing tools in the clinical and regulatory context? (In this discussion, we will consider ML algorithms applications in the clinics or in the lab and not the more abstract knowledge-generating tasks such as the mapping of the Cancer Genome Atlas described previously. This latter question is less a methodological one and more of an epistemological one, having to do with the logic of induction and scientific discovery: see, for instance, López-Rubio and Ratti [10].)

In order to answer this question, it is helpful to consider the similar situation of Bayesian statistical methods for the analysis of clinical trial results [13]. Bayesian statistics has been presented as an alternative to classical analysis of trial results using *p*-values and confidence intervals. Bayesian methods promise to have superior performance as compared to classical ones in specific tasks (such as interim analysis or multifactor analysis); however, when it comes to replacing the gold standard, better performance is only a part of the picture. The traditional methodology for trial result analysis has the *trust* of the medical community, that has learned how to interpret and use it; it is mature, with a proven *track record* of applications; and finally, it is accepted by regulatory bodies as a tool that warrants objectivity and *accountability* in the trial review process.

In order to be routinely accepted and used in hospitals, ML software for diagnosis and treatment has to attain a similar level of compliance along these three criteria: trust, or the acceptance and reliance on the technique by the medical professionals who will be using it; track record, or the level of adoption and maturity of the technology; and finally accountability, or the possibility to assign responsibility of the decisions taken by ML systems. Let us see each in turn.

Firstly, concerning trust, we have seen that it can be built through successful applications of the technique; however, reliability and external validity of ML algorithms are often at issue in biomedical applications. Indeed, many review articles in the field denounce poor predictive performance as a common problem in the works they examine (see, for instance, Cruz [5] or Kourou *et al* [15]). We should not conclude that ML techniques are unreliable; there are several adjustments and methodological tools that can be employed to correct bias, reinforce external validity and enhance predictive power. What is true, however, is that ML applications to the biomedical field are still too young for taking them for granted. To make another comparison, techniques such as gene-expression analysis or microarray are at this point in time so standardised that, in evaluating their results, one would hardly question the procedure followed or the methods used. Not so for ML, where it is legitimate to worry about possible bias or overfitting in training of the model, and trust has to be established on a case-by-case basis.

The second criterion to be examined is the track record of ML applications. At present, ML is still at a stage of early development where different avenues of application are explored and the technique is put to use in different ways in order to identify successful use cases. We are still far from a situation where a ML solution becomes the standard, go-to method for a particular diagnostic or clinical task. However, as a novel technique matures and becomes more reliable, its intrinsic advantages will increase its adoption, and thus consolidate its status. For instance, there can be no doubt about the strength of ML algorithms in tasks such as visual diagnosis; it is likely that, in time, the presently experimental ML solutions for automated diagnosis will evolve into standard, commercial applications and the same could happen for any other of the applications we have seen in this review.

This brings into consideration the last criterion on our list: accountability. The inevitable errors of a digital system for automated diagnosis or for automated therapy choice will have grave consequences for which it must be possible to adjudicate responsibility (for a discussion of the ethical aspects of this scenario see Grote and Berents [9]). In the beginning of the previous Section, we observed that for this reason it is likely that the diagnostic or treatment decision will still ultimately be taken by the human doctor. If so, what may be the consequences when the recommendation given by the algorithm turns out to be erroneous or if it contradicts the clinician’s own opinion?

On one hand, we should consider that the outcome of the ML algorithm comes with a certain confidence and a certain possibility of error. In this it is similar to other diagnostic or therapeutic aids, such as various kinds of laboratory analysis. What is different, though, is that the true accuracy of ML algorithms may be difficult to determine. Clinicians may be opposed to taking responsibility for an indication that opposes their own, also considering that the evidence and reasoning behind the ML result will mostly remain inaccessible. It seems that what is required is a model where liability is shared, like the situation where a laboratory may be held legally responsible in the case of erroneous test results [6]. In the case of a ML algorithm, their share of responsibility may be assigned to the software house who built them; however, this liability is likely to discourage many firms from investing in this endeavour. In conclusion the problem of accountability may be the hardest to address, and therefore it may be the most relevant factor in delaying the widespread adoption of ML techniques in such contexts.

## Conclusion

ML is a set of techniques that promise to greatly enhance our data-processing capability. In the field of oncology, ML presents itself with a wealth of possible applications to the research and the clinical context, with examples spanning from diagnosis to prognosis to treatment modulation.

When considering such applications, it is important to maintain a balanced perspective: ML can provide new and powerful tools to address the hard problems that researchers and clinicians are daily confronted with in this field; however, ML has the potential to be error-prone or vulnerable to built-in bias in the same manner as other tools in medical research and practice.

As a consequence, it is necessary to avoid premature over-reliance on ML algorithms: trust in this technology has to be built one step at a time, based on its capability to make useful and correct predictions. The introduction of ML techniques to the clinical routine has to follow the right pace in order to enhance a widespread adoption of ML and make it possible to reap its benefits for patients and for the advancement of oncological practice.

## Conflicts of interest

None stated. The author is an independent researcher and does not receive funding.
